# Fourier Transform Infrared Spectroscopic Imaging and Multivariate Regression for Prediction of Proteoglycan Content of Articular Cartilage

**DOI:** 10.1371/journal.pone.0032344

**Published:** 2012-02-16

**Authors:** Lassi Rieppo, Jarno Rieppo, Jukka S. Jurvelin, Simo Saarakkala

**Affiliations:** 1 Department of Applied Physics, University of Eastern Finland, Kuopio, Finland; 2 Department of Clinical Neurophysiology, Kuopio University Hospital, Kuopio, Finland; 3 Institute of Biomedicine, Department of Anatomy, University of Eastern Finland, Kuopio, Finland; 4 Iisalmi Hospital, Iisalmi, Finland; 5 Department of Diagnostic Radiology, Institute of Diagnostics, University of Oulu, Oulu, Finland; Universitat Rovira i Virgili, Spain

## Abstract

Fourier Transform Infrared (FT-IR) spectroscopic imaging has been earlier applied for the spatial estimation of the collagen and the proteoglycan (PG) contents of articular cartilage (AC). However, earlier studies have been limited to the use of univariate analysis techniques. Current analysis methods lack the needed specificity for collagen and PGs. The aim of the present study was to evaluate the suitability of partial least squares regression (PLSR) and principal component regression (PCR) methods for the analysis of the PG content of AC. Multivariate regression models were compared with earlier used univariate methods and tested with a sample material consisting of healthy and enzymatically degraded steer AC. Chondroitinase ABC enzyme was used to increase the variation in PG content levels as compared to intact AC. Digital densitometric measurements of Safranin O –stained sections provided the reference for PG content. The results showed that multivariate regression models predict PG content of AC significantly better than earlier used absorbance spectrum (*i.e.* the area of carbohydrate region with or without amide I normalization) or second derivative spectrum univariate parameters. Increased molecular specificity favours the use of multivariate regression models, but they require more knowledge of chemometric analysis and extended laboratory resources for gathering reference data for establishing the models. When true molecular specificity is required, the multivariate models should be used.

## Introduction

Articular cartilage (AC) is a highly specialized tissue that covers the ends of long bones. The major constituents of AC are water, type II collagen, proteoglycans (PGs) and cells, *i.e.*, chondrocytes [1]. The unique functional properties of AC are achieved by its inhomogeneous composition and structure [2]. Detailed information on the spatial distribution of the biochemical constituents is needed for several reasons. For example, it has been proposed that the mechanical properties of AC could be estimated solely based on the biochemical composition by using sophisticated mathematical models [3]. Furthermore, in order to clarify the biochemical changes of AC in early phase of osteoarthritis, highly specific imaging techniques are required.

Vibrational spectroscopy consists of a collection of different techniques including mid-infrared spectroscopy (commonly referred to as infrared (IR) spectroscopy or Fourier transform Infrared (FT-IR) spectroscopy), near infrared spectroscopy (NIR) and Raman spectroscopy. Vibrational spectroscopic techniques are typically considered to be complementary to each other. NIR has the advantage of a better penetration into a sample, enabling measurements with little or no sample preparation. On the other hand, NIR lacks the molecular selectivity of FT-IR and Raman spectroscopy. Since water strongly absorbs mid-IR light, thus hindering the analysis of more interesting components, FT-IR studies must be usually conducted using dry samples, whereas NIR and Raman spectroscopic measurements can be done also from aqueous samples. The weakness of Raman spectroscopy is that Raman scattering is very weak compared to elastically scattered light resulting in a much worse signal-to-noise ratio in Raman spectroscopy than in IR spectroscopy. Therefore, each technique has its advantages and disadvantages compared to the others.

FT-IR spectroscopic imaging is a technique that is capable of producing biochemical images from histological sections, *i.e.*, images that show the spatial variation of biochemical components. Traditionally, univariate analyses have been used in cartilage FT-IR research, *e.g.*, by calculating the area of an absorption peak from the measured spectra [4]. This approach is limited by the fact that one can rarely find a specific peak for any tissue component directly from the absorption spectrum, since significant overlap exists between different absorption peaks [5]. The reason for spectral overlapping arises from the fact that biological tissues have very similar biochemical composition since all proteins are built from the same amino acids. Therefore, biochemical differences between tissues are mainly due to different protein folding and side chain substitutions. As the detected changes in spectra between different tissue types are small, it is very difficult to obtain contrast between different tissue types from histological section when univariate-based methods are used. As a step forward, derivative spectra can be used for better separation of the overlapping peaks but it cannot completely solve the problem. The use of derivative spectra also requires a high signal-to-noise-ratio since noise is greatly amplified in differentiation process [6]. In general, univariate techniques abandon lots of information since only one variable at a time is investigated. Univariate analysis requires an intuitive assumption, or *a priori* information, of the spectral feature or the wavenumber range that exhibits the adequate specificity for the studied chemical compound. Therefore, the technique is limited and the collected chemical information cannot be used as effectively as possible.

Multivariate analysis techniques use several independent variables simultaneously when the biochemical composition of the tissue is analyzed from FT-IR measurements. Multivariate techniques can use the entire collected spectral information for the analysis. *A priori* information of the specificity of spectral regions is not required, since a wide spectral region is usually used. Multivariate techniques have been shown to be more powerful than univariate techniques [7]. Principal Component Regression (PCR) and Partial Least Squares Regression (PLSR) are popular chemometric methods for quantitative analyses [7]. Both methods construct new variables that are used as regressors. The difference between PCR and PLSR is that PCR constructs variables to explain variance in measured spectra, while PSLR constructs variables to explain co-variance between the spectra and predicted information. Therefore, variables of PLSR may contain more accurate information on the predicted content. When a PCR or a PLSR model is built, one needs to calibrate it against the reference information on, *e.g.*, the biochemical composition of the samples. This calibration procedure requires a large dataset of spectral measurements and counterparting reference measurement values. Data collection for calibration can be time consuming, but once the model is built and properly validated the analysis of large data sets can be done fast and reliably.

In the FTIR research of articular cartilage and osteoarthritis, two studies have reported that area of carbohydrate region of infrared absorption spectrum correlates with histologically and biochemically determined PG content in tissue engineered cartilage [8], [9]. However, these studies used only bulk values of tissue engineered cartilage, which contains significantly less collagen than native cartilage [10]. It has also been reported that synthesized glycosaminoglycans are significantly different from those found from native cartilage extracellular matrix [11], [12]. Therefore, these previous results are not necessarily directly applicable to normal articular cartilage. Recent studies have shown that current univariate-based methods lack the specificity for PG molecules especially at the superficial layer of AC [5], [13], and the existing univariate methods cannot reach the specificity level of the biochemical methods [5]. Increased specificity for FT-IR analysis is needed before the technique can be reliably used for cartilage research and before it can partially replace the existing biochemical methods. Recently, PCR was used to predict collagen and PG concentrations in bovine nasal cartilage [14]. Furthermore, several multivariate methods were used to analyze the biochemical composition of AC from Raman spectroscopic data [15]. However, PLSR has not yet been used in FT-IR studies for quantitative compositional analysis of AC.

The aim of this study was to introduce PLSR modelling in prediction of the PG content of AC. Both intact and enzymatically degraded steer AC samples was used, and the potential of PLSR modelling, as well as of earlier used univariate-based analysis methods and PCR, to predict the biochemical reference values was clarified. As PLSR modelling has not been previously used for this research problem, we hypothesized that it improves specificity for PG molecules beyond the earlier used univariate analysis methods.

## Materials and Methods

### Sample preparation

Knee joints of 1-3-year-old steers (*n* = 16) were obtained from a local abattoir (Atria Oyj, Kuopio, Finland). Osteochondral plugs (*d* = 13 mm) were prepared from the lateral upper quadrants of the patellae. Samples were divided into two groups of eight samples. Samples of the first group were modified with chondroitinase ABC enzyme (concentration 30 U/ml at 37°C for 44 h) for removal of superficial PGs. Samples in the second group served as controls. Subsequently, the samples were fixed with 10% formalin, decalcified, dehydrated and embedded in paraffin as described earlier [16]. 5-µm-thick vertical sections were cut with a microtome and transferred onto standard objective slides. Paraffin was dissolved with xylene prior to transferring the sections onto 2-mm-thick ZnSe windows for the FT-IR spectroscopic imaging measurements.

### FT-IR spectroscopic imaging

Measurements were conducted with the Perkin Elmer Spotlight 300 FT-IR imaging system (Perkin Elmer, Shelton, CO, USA) in transmission mode using spectral resolution and pixel resolution of 4 cm^−1^ and 25 µm, respectively. Eight repetitive scans per pixel were averaged. The imaging system and the sample box were purged with CO_2_-free dried air during the measurements to standardize the measurement conditions (Parker Balston, Haverhill, MA, USA). Data was collected from cartilage surface to cartilage-bone interface using a 400-µm-wide region of interest.

### Reference measurements (Digital densitometry)

PG content and spatial PG distribution was estimated indirectly using digital densitometry (DD). As Safranin O is cationic, it binds to the negatively charged glycosaminoglycans, and therefore the staining intensity follows the PG distribution of the sample. Stain absorption can be quantitatively measured with microscope coupled with CCD-camera. Optical density (OD) of safranin O is directly related to the amount of PGs in the sample. DD of Safranin O is a well validated reference method for determination of spatial distribution of negatively charged glycosaminoglycans in AC [17], [18]. The main advantage of DD over traditional biochemical methods is that PG content from superficial tissue to deep cartilage can be determined from a single section without sectioning the sample into multiple subsamples. System consists of a Leitz Orthoplan light microscope (Leitz Wetzlar, Wetzlar, Germany) using monochromatic light (λ = 492±5 nm) and a peltier-cooled 12-bit CCD camera (CH250, Photometrics, Tucson, AZ, USA). System was calibrated with neutral density filters (Schott, Mainz, Germany) to cover a range from 0 to 3 OD values. Multiple 3-µm-thick sections were cut from each sample. Paraffin was dissolved from the sections with xylene, and the sections were stained with Safranin O. Three randomly selected sections were measured and averaged from each sample to reduce the effect of variable section thickness. The results were averaged in transverse direction (parallel to the surface) to obtain depth-wise PG distribution of AC [16].

### Data pre-processing

Second derivative spectra were calculated from FT-IR data by using Savitzky-Golay algorithm (7 smoothing points). Since only the depth-wise information was studied, both the FT-IR data and safranin O data were averaged in transverse direction (*i.e.* parallel to the surface). However, direct comparison was not possible, since the pixel size in DD measurements is smaller than in FT-IR measurements (∼5 µm vs. 25 µm). Therefore, the depth-wise safranin O profiles were resampled to obtain the same number of data points for both techniques. This was possible, because full cartilage thickness was measured with both techniques. Cartilage surfaces and cartilage-bone junctions between the FT-IR and safranin O data were first manually matched. Subsequently, the depth-wise safranin O profiles were resampled in order to get equal number of safranin O data points to the number of FT-IR spectra obtained from the same sample. After the resampling, each FT-IR spectrum had one reference value that indicated the PG content in the corresponding location of the sample. This enabled the direct comparison between the FT-IR parameters and the DD reference measurements.

### Partial Least Squares Regression (PLSR) and Principal Component Regression (PCR)

Data set was formed so that all PG concentration levels were evenly represented. All data covering OD values of 0–1.5 OD (144 data points) were included entirely into the used data set. Since OD values from 1.5 to 2.3 formed the majority (838 out of 932 data points) of the collected data, only part of this data was included in the data set. Consequently, 50 data points from each OD value ranges 1.5–2.0 OD, 2.0–2.15 OD and >2.15 OD were randomly selected to the data set. Altogether, the data set consisted of 294 data points.

Spectral regions of 1000–1440 cm^−1^ and 1480–1700 cm^−1^ were used in PLSR and PCR models. The region of 1440–1480 cm^−1^ was not used, since some sections still contained traces of paraffin, which shows strong absorption bands in this region. Number of components for the models was chosen based on the root-mean-square error of cross validation (RMSECV) of the data set. In leave-one-out cross validation, one sample in turn is removed from the training data to be used as a validation data. Predicted values of each sample are then stored and finally compared to reference data [7], [19]. The performance of final PLSR and PCR models was evaluated by Pearson's correlation coefficient obtained by comparing the model predictions after cross validation with the corresponding OD values.

One enzymatically modified sample and one control sample were left completely out of the multivariate models as a purpose of using these samples when demonstrating the use of validated PLSR model in imaging studies.

### Univariate FT-IR analyses

Previously, PG content of AC has been analyzed from absorption spectrum by calculating the integrated area of carbohydrate region (984–1140 cm^−1^) or ratio of carbohydrate region to amide I peak (1584–1720 cm^−1^) [4], [9], [20]. Also second derivative spectra contain PG-related information, *e.g.* the peaks located at 1062 cm^−1^ [5], [21], [22] and at 1374 cm^−1^ [23]. As a purpose of comparison, these parameters were also calculated and compared with the results of PLSR model. Spectral data was baseline corrected using an offset correction prior to data analyses. Values of univariate parameters were converted to OD values by using the linear regression equations, and thereafter the root-mean-square errors (RMSEs) were calculated. All data analyses were performed using Matlab (Ver. R2007b, MathWorks Inc., Sherborn, MA, USA).

### Comparison between univariate and multivariate analysis methods

FT-IR-based PG parameters were initially compared with the reference PG distributions by calculating Pearson's correlation coefficients. The statistical difference between the correlation coefficients was tested by comparing the elements of the correlation matrices as described by Steiger [24]. Since multiple comparisons between correlation coefficients were involved, Bonferroni correction was applied to the obtained significance values (*N* = 7 comparisons, the level of significance: *p*<0.05/N = 0.007). Furthermore, different analysis methods were compared to each other by analyzing the relative prediction errors among different OD values (absolute prediction error/predicted value).

## Results

**Figure 1:**
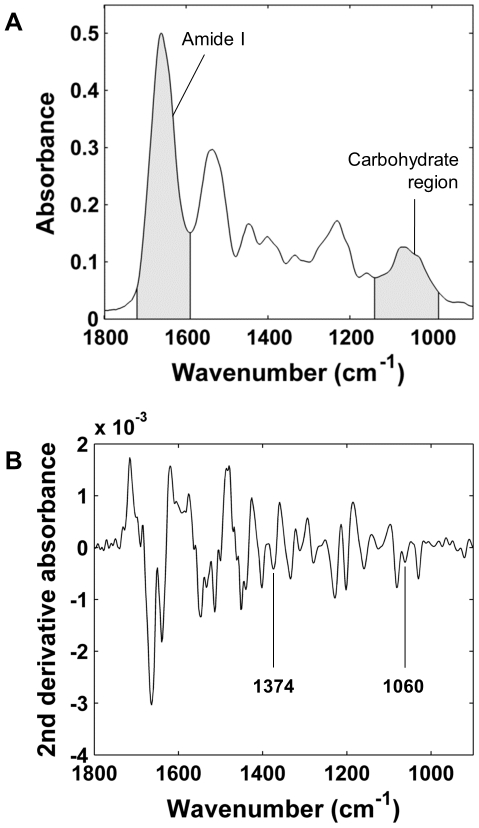
Mean absorption spectrum and second derivative spectrum of the data set. A) Mean infrared absorption spectrum of AC and B) mean second derivative spectrum of AC.

**Figure 2:**
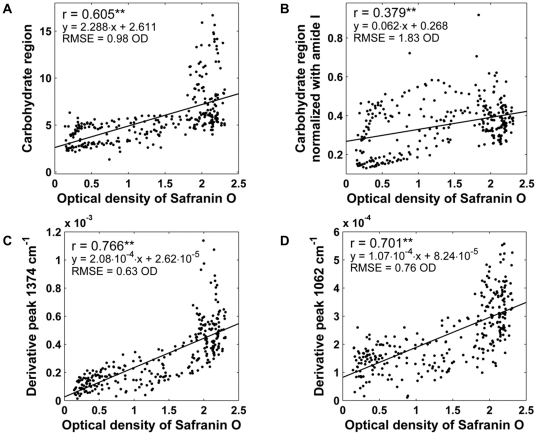
Scatter plots between optical density of safranin O and different univariate FT-IR PG parameters. A) Integrated area of carbohydrate region (984–1140 cm^−1^). B) Integrated area of carbohydrate region normalized with amide I (1584–1720 cm^−1^). C) Intensity of ^2nd^ derivative peak located at 1374 cm^−1^. D) Intensity of 2^nd^ derivative peak located at 1062 cm^−1^. Statistical significance of *p*<0.001 is indicated by two asterisks (**).

Mean infrared absorption spectrum of AC and mean second derivative spectrum of AC are shown in Figure 1. Integrated area of carbohydrate region showed positive correlation agaist reference distribution (*r* = 0.605, *p*<0.001, RMSE = 0.98 OD) (Figure 2A). Normalization of carbohydrate region with amide I weakened the correlation (*r* = 0.379, *p*<0.001, RMSE = 1.83 OD) (Figure 2B). The difference between the correlation coefficients was statistically significant (*p*<0.007).

The results were improved by using the second derivative spectra. The intensity of the second derivative peak at 1374 cm^−1^ correlated slightly better with the reference PG data (*r* = 0.766, *p*<0.001, RMSE = 0.63 OD) (Figure 2C) than the intensity of the second derivative peak 1062 cm^−1^ (*r* = 0.701, *p*<0.001, RMSE = 0.76 OD) (Figure 2D). The difference between the correlations reached the level of statistical significance (*p*<0.007). Furthermore, both second derivate PG parameters were better predictors of PG content than the area of carbohydrate region (*p*<0.007) as judged from correlations with the reference distribution as well as from RMSE values.

**Figure 3:**
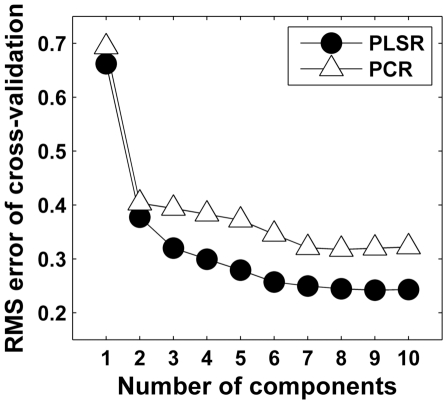
Root-mean-square error of cross-validation (RMSECV) with different number of components for PLSR and PCR. Based on RMSECV, 7 components were selected for both models as RMSECV did not decrease significantly with additional components.

**Figure 4:**
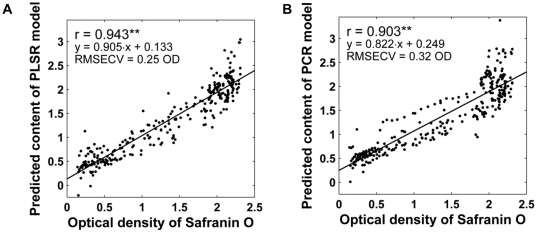
Scatter plots between optical density of safranin O and FT-IR multivariate models. A) Predicted PG content obtained from PLSR model. B) Predicted PG content obtained from PCR model. Statistical significance of *p*<0.001 is indicated by two asterisks (**).

RMSECV with different number of PLSR and PCR components are shown in Figure 3. Based on RMSECV, 7 components were selected for both models as RMSECV did not decrease significantly with additional components. PLSR model showed a high positive correlation with the reference PG data (*r* = 0.943, *p*<0.001, RMSECV = 0.25 OD) (obtained by cross-validation) (Figure 4A). PCR model also produced good results (*r* = 0.903, *p*<0.001, RMSECV = 0.32 OD) (obtained by cross-validation) (Figure 4B). Both PCR and PLSR model performed better than the best univariate parameter (second derivative peak 1374 cm^−1^) (*p*<0.007) as judged from correlations with the reference PG distribution as well as from RMSE values. Further, PLSR was proven to be the best predictor for PG content as the difference between PLSR and PCR was statistically significant (*p*<0.007).

**Figure 5:**
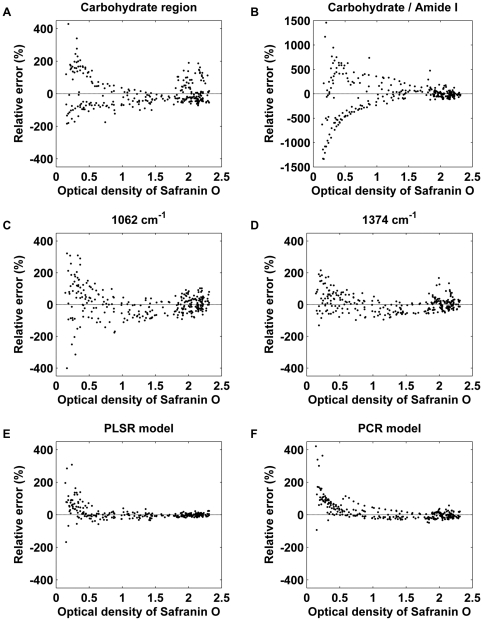
Relative prediction errors of the different FT-IR PG parameters. A) Integrated area of carbohydrate region (984–1140 cm^−1^) (mean error: ±35.9%). B) Integrated area of carbohydrate region normalized with amide I (1584–1720 cm^−1^) (mean error: ±107.8%). C) Intensity of 2^nd^ derivative peak located at 1062 cm^−1^ (mean error: ±33.0%). D) Intensity of ^2nd^ derivative peak located at 1374 cm^−1^ (mean error: ±20.6%). E) PLSR model (mean error: ±10.7%). F) PCR model (mean error: ±16.9%). Note that plot B has a different scale on y axis.

Accuracy of the different analysis approaches were further investigated by analysing the relative prediction error for each method at different OD values. Carbohydrate region –based and second derivative –based univariate parameters showed larger relative error throughout the whole OD range as compared to multivariate models (Fig. 5). Carbohydrate parameter had non-random variation with high PG values and some obvious clustering was evident where parameters overestimated the actual amount of PGs (Fig. 5 A). Amide I normalization weakened the results especially at low OD values (Fig. 5 B). Both second derivative parameters had rather uniform and random variation at all OD values and no systematic deviation was detected (Figs 5 C and D). However, the peak 1062 cm^−1^ showed higher relative error than the peak 1374 cm^−1^ or the carbohydrate region at low OD values. It should be noted that the multivariate models had smaller variation throughout the whole OD range compared to univariate parameters and gave consistent results also at the large OD values (Figs 5 E and F).

**Figure 6:**
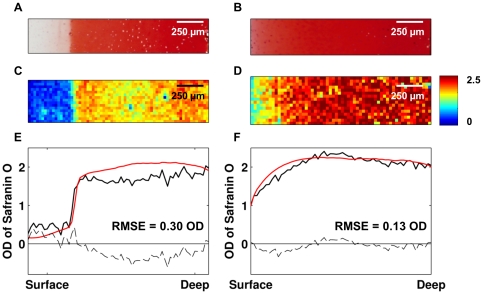
An enzymatically degraded (left) and a normal (right) sample. Safranin O –stained sections are shown in A and B. Predicted PG contents by the PLSR model are shown in C and D. The loss of superficial PGs is easily seen in C. Depthwise distribution profiles of the same samples analyzed by the PLSR model (black) and by digital densitometry (red) are shown in E and F. The difference between the predicted and measured values are marked with dashed lines in E and F. RMSEs were 0.30 OD and 0.13 OD for the enzymatically modified sample and intact sample, respectively.

Suitability of the PLSR model for imaging studies was tested by analyzing 2D FT-IR images of two additional bovine samples (not included in the PLSR model training data). The results were compared with the safranin O –stained parallel sections (Figure 6). PLSR model was shown to give consistent results with safranin O staining and the model detected accurately the partial PG depletion generated with the chondroitinase ABC enzyme.

## Discussion

Our results show the feasibility of the application of PLSR in the FT-IR spectroscopic imaging data analysis in AC. The original hypothesis was confirmed in the study, as the PLSR model clearly outperformed the previously used univariate analysis methods for the prediction of the spatial PG content of AC. The approach of the present study enables direct comparison of different analysis methods since the parameters were derived from the same datasets, *i.e.*, the potential sources of errors, related to sample preparation and measurement conditions, were identical for each parameter. The obtained results are promising and clearly show the potential of multivariate analysis techniques for overlapping spectral data. PCR, which was recently used to predict collagen and PG concentrations of bovine nasal cartilage [14], also performed well although the results were a little worse than with PLSR. This was expected, as PCR utilizes only the measured spectral data when new variables are constructed, while PLSR also uses the predicted information.

In earlier studies, the carbohydrate region has been found to correlate with biochemically determined PG content. These studies have used bulk PG concentrations of tissue engineered cartilage [8], [9]. In this study, steer AC was used. Further, DD of Safranin O stained parallel sections was chosen as the reference method for PG content, because DD enables the determination of PG content in different layers of AC from a single cartilage section. Collagen-to-PG ratio varies throughout the cartilage depth, which makes it difficult to determine either collagen or PG content by univariate methods. These differences might explain why univariate methods seem to perform worse in our study than in earlier studies. The findings of the present study indicate that the largest absolute prediction errors occur in the deep tissue, where the PG and collagen contents are high. In contrast, it was earlier found that the specificity of univariate-based PG parameters is limited in the superficial tissue of normal and especially of osteoarthritic human AC, while they work reasonably well in the middle and deep layers [13]. The most probable explanation for this discrepancy is that different species were used in the studies. PG and collagen distribution profiles are significantly different in bovine or porcine tissue than in human tissue [25], [26], [27]. In all mammalian cartilage tissues, PG concentration rapidly increases after the superficial layer and relatively uniform high PG amounts are obtained from various depths of tissue [16], [28]. On the other hand, human AC has remarkably less depth-wise variation in collagen content than bovine or porcine AC. In bovine and porcine, collagen content increases monotonically from the middle layer to the deep layer, creating a variable collagen-to-PG ratio for most parts of the cartilage. This probably explains the difficulties in predicting the largest PG values in the current study using steer AC.

Lack of specificity with univariate parameters, calculated from the raw spectra, manifests as an increased variation in the predicted high PG values from the middle and deep layers, as seen with the present results (Figs 2 and 5). Second derivative-based univariate parameters showed smaller systematic estimation errors than the carbohydrate region-based parameters. However, second derivative parameters are more vulnerable to random noise associated with the measurement. Multivariate analysis has a theoretical premise to work better than the univariate parameters in situations with a variable collagen-to-PG ratio, although absolute errors are still slightly higher with high PG values (Fig. 3). However, the relative error is actually quite small at high PG values for the multivariate models (Fig. 5). Based on the relative error, the smallest PG values seem to be difficult to predict accurately. This can be partly explained by the fact that the relative error easily becomes large when the predicted values are close to zero. Ideally, the measurement error consists only of random noise. The amplitude of random noise is independent of the measured concentration levels. Therefore, a larger relative error is observed at smaller OD values no matter what kind of analysis technique is used. Since the earliest osteoarthritic changes include loss of PGs in the superficial tissue [29], and as the superficial tissue PG content plays an important role for biomechanical behavior [16], [30], it is important that the analysis method works well also with the low end of the PG levels (superficial tissue). It is demanding to determine small concentrations accurately. Multivariate techniques, such as PCR and PLSR, filter some of the random noise by rejecting the insignificant, noise-related components. Random noise could be further decreased by increasing the number of scans per pixel, which would result in more accurate determination of PG content in terms of relative error.

Alternative explanation for the difficulties in the deep cartilage could be found from the reference technique used, which measures the sulphated glycosaminoglycans of PGs. The second derivative peak at 1062 cm^−1^ has been associated not only with sulphates [22], but also with C-O vibrations [21], [31]. Therefore, non-sulphated glycoproteins might partly explain the overestimation of high concentration levels. On the other hand, the second derivative peak at 1374 cm^−1^ is associated with the CH_3_ group [23], which is found in all glycosaminoglycans, and therefore is not directly related to sulphates. The slightly weaker correlation of peak 1062 cm^−1^ compared to peak 1374 cm^−1^ might be also due to its weak intensity, which brings more random variation to the data. When a wide part of the carbohydrate region is used, limitations can be seen more clearly, since the carbohydrate region also contains vibrations related to collagen. Normalization of the carbohydrate region with amide I seemed to even worsen the results. Similar limitations in specificity of carbohydrate-based parameters were also reported in the earlier study using human AC samples [13]. Consequently, the limited specificity of univariate parameters should be taken into account in all future FT-IR investigations of AC.

In this study, some uncertainty arises from the fact that only one section per sample was measured with FT-IR. It is known that significant section-to-section variation in section thickness exists even when sections are cut in the same session [32]. This brings random variation in the FT-IR data between the samples. A random variation in the section thickness most likely reduces the obtained correlation levels of the FT-IR parameters. The problem is probably more significant with univariate parameters, since multivariate techniques can partly take into account the thickness variation based on the total absorbance of the investigated spectral region. In the future, multiple sections should be measured and the average results reported, or alternatively the section thickness differences should be reduced with reference sample normalization [32].

An obvious advantage of the PLSR is that it is not limited by the user's ability to pinpoint a specific region to obtain an adequate specificity for a desired tissue component. Spectral peaks are wide in biospectroscopy, and very seldom can a separate well-defined peak be found to represent a single molecule. Thus, in general, molecular-level specificity cannot be established with univariate-based methods. Multivariate techniques, on the other hand, are limited mainly by the reference data, the reference technique and FT-IR spectroscopic measurement itself. Collected spectral information can be used more effectively with multivariate techniques since all the collected information can be included in the model. Furthermore, the PLSR model is built using predefined algorithms and accurate *a priori* information of the specific spectral peaks is not needed.

Collection of the reference data for a multivariate model can be very time consuming and it requires laboratory resources. In this study, Safranin O –stained sections were used as a reference. The main reason for this was the ability of Safranin O –staining to produce several reference values from a single specimen. Pixel by pixel data was not used for comparison as the reference measurements were not performed from the exact same sections as the FT-IR spectroscopic measurements. Instead, the data was averaged in the transverse direction which also reduced effectively random noise. Optical density of Safranin O in the determination of PG content of AC has earlier been validated by two separate reference techniques. Strong correlations between the optical density of Safranin O and the reference techniques were obtained in both cases (*r* = 0.96 with thin layer chromatography and *r* = 0.995 with Na^+^ tracer). Safranin O traces the negative charge in AC that is primarily caused by the sulphated glycosaminoglycans. Therefore, the optical density of Safranin O can be regarded as a valid reference technique for this study. The biggest source of error in DD is the variable section thickness, which brings random variation to the data. This was reduced by measuring three sections per sample and averaging the results.

In the future a multivariate model for the prediction of AC composition should be built using the gold standard as the references, *i.e.*, biochemical methods. Although building the PLSR model is time consuming, the subsequent application of the model is fast and reliable. From the theoretical point of view, after a proper validation process multivariate spectral analysis may allow quantitative spatial PG and collagen determination from histological sections with similar specificity as the current biochemical techniques.

When considering the validation of the PLSR model, it is noteworthy that sample processing may affect the outcome of the model. In the present study, formalin-fixed samples were used. The model validated with formalin-fixed samples may not perform equally well with cryosections, also generally used in FT-IR spectroscopic imaging studies, since fixation may alter the spectra. This aspect should be studied more closely. Possibly, different models should be built for cryosections and formalin-fixed sections.

The use of univariate analysis for the determination of PG and collagen contents of AC is favored by the simplicity of the analysis, as the analysis can easily be performed with any available software. The specificity of univariate analysis for PGs can be improved by the application of second derivative spectroscopy at the expense of amplification of noise. However, the users should be aware of the limitations of univariate analysis. Present results indicate that the multivariate techniques should be preferred for compositional studies of AC when true biochemical specificity is desired. Application of the FT-IR spectroscopic techniques for cartilage research is still hindered by the lack of parameter specificity for PGs and collagen. Chemical imaging itself would offer a great potential for characterization of AC since the traditional biochemical techniques have only limited capabilities for characterization of the spatial distribution of tissue constituents. The application of multivariate techniques is a step towards true molecular imaging of AC.
